# Efficient pathway for men fertility preservation in testicular cancer or lymphoma: a cross-sectional study of national 2018 data

**DOI:** 10.1186/s12610-023-00209-8

**Published:** 2023-12-12

**Authors:** Ségolène Prades, Sarah-Lyne Jos, Jacqueline Saïas-Magnan, Louis Bujan, Florence Eustache, Oxana Blagosklonov, Eric Lechevallier, Florence Brugnon, Vanessa Loup-Cabaniols, Dorian Bosquet, Marie Prades, Bérengère Ducrocq, Céline Chalas, Sandrine Giscard-d’Estaing, Anne Mayeur, Isabelle Koscinsky, Françoise Schmitt, Aline Papaxanthos-Roche, Marius Teletin, Emmanuelle Thibault, Damien Beauvillard, Sophie Mirallie, Béatrice Delepine, Annie Benhaim, Pascale May-Panloup, Ségolène Veau, Cynthia Frapsauce, Patricia Fauque, Régis Costello, Nathalie Rives, Catherine Metzler-Guillemain, Jeanne Perrin, the CECOS network

**Affiliations:** 1 CECOS/Laboratory of Reproductive Biology, La Conception University Hospital, 13385 Marseille, France; 2 DEFE (Développement Embryonnaire, Fertilité, Environnement) INSERM, Universités Montpellier Et Toulouse 3, CECOS Hôpital Paule de Viguier, CHU de Toulouse, 1202, Toulouse, France; 3 CECOS, Site Jean Verdier, Hôpitaux Universitaires Paris Seine-Saint-Denis, Bondy, France; 4 Genomics, Epigenetics and Physiopathology of Reproduction, Institut Cochin, Inserm U1016, Paris, France; 5 Service de Biologie Et Medecine de La Reproduction, Cryobiologie-CECOS, CHRU Jean Minjoz, Besançon, France; 6 Service d’Urologie et Transplantation Rénale, Aix-Marseille Université, Assistance Publique Hôpitaux de Marseille, Marseille, France; 7 1240 IMOST, INSERM, Clermont Ferrand, France; 8 Service AMP CECOS, CHU Clermont Ferrand, Clermont Ferrand, France; 9 CECOS Languedoc Roussillon, MONTPELLIER Hôpital Arnaud de Villeneuve, Montpellier, France; 10 Service de Médecine Et Biologie de la Reproduction – CECOS-CHU Amiens Picardie - Site Sud, Amiens, France; 11 Service de Biologie de La Reproduction-CECOS, Hôpital Tenon (AP-HP), Sorbonne-Université, 75020 PARIS, France; 12 Institut de Biologie de la Reproduction - CECOS Hôpital Calmette, CHU de Lille, Lille, France; 13 Laboratoire de Biologie de la Reproduction-CECOS, Assistance Publique-Hôpitaux de Paris.Centre Université Paris-Cité, GHU Cochin, Paris, France; 14 Biologie de La Reproduction, U1208, Hospices Civil de Lyon, HFME, Inserm, Bron, France; 15 Faculté de Médecine Lyon Sud, Université Claude Bernard, Oullins, France; 16 Reproductive Biology Department, CECOS, Paris-Saclay University, Antoine-Béclère Hospital, APHP, Clamart, France; 17 NGERE (Nutrition Génétique Et Exposition Aux Risques Environnementaux) INSERM 1256 Université de Lorraine, 10 Avenue de La Forêt de Haye, 54505 Vandoeuvre Les Nancy, France; 18 Laboratoire de Biologie de La Reproduction, Hôpital Saint Joseph 26 Boulevard de Louvain, 13008 Marseille, France; 19 CECOS ALSACE Mulhouse Groupe Hospitalier, de La Région de Mulhouse Et Sud Alsace, Mulhouse, France; 20 Biology of Reproduction-CECOS Laboratory, University Hospital of Bordeaux, Bordeaux, France; 21 Institut de Génétique Et de Biologie Moléculaire Et Cellulaire (IGBMC), Université́ de Strasbourg, France-LBDR-CECOS, Hôpitaux Universitaires de Strasbourg (HUS), , Strasbourg, France; 22 Laboratoire de Biologie de la Reproduction – CECOS Hôpital L’Archet 2 - CHU de Nice, Nice, France; 23 CHU Brest, AMP CECOS, Brest, France; 24 Service de Médecine Et Biologie de La Reproduction, CHU Nantes, France; 25 Service de Biologie de La Reproduction Reims - Pôle de Biologie Médicale Et Pathologie, Reims, France; 26 Service de Biologie de La Reproduction Coordinatrice Clinico-Biologique du Centre d’AMP du CHU de Caen Pôle de Biologie-CHU, Caen, France; 27 Biologie de La Reproduction, Centre Hospitalier Universitaire & Univ Angers, INSERM, CNRS, MITOVASC, Equipe MitoLab, SFR ICAT, 49000 Angers, France; 28 Service de Biologie de La Reproduction-CECOS, CHU Rennes - Hôpital Sud, Rennes, France; 29 Service de Médecine Et de Biologie de La Reproduction-CECOS, CHRU Bretonneau, Tours, France; 30 Burgundy University, INSERM 1231, Dijon, France; 31 Dijon University Hospital, Biology of Reproduction-CECOS Laboratory, Dijon, France; 32 Service d’Hématologie Et Thérapie Cellulaire, Hôpital La Conception, Assistance Publique Des Hôpitaux de Marseille, 147 Boulevard Baille, 13005 Marseille, France; 33 NorDiC UMR 1239, team “Adrenal and Gonadal Pathophysiology”, Biology of Reproduction-CECOS Laboratory, Univ Rouen Normandie, Inserm, Rouen University Hospital, Rouen, France; 34 Aix Marseille Univ, INSERM, MMG, UMR_S1251, Marseille, France; 35 Aix Marseille Univ, Avignon Université, CNRS, IRD, IMBE, Marseille, France

**Keywords:** Chemotherapy, Radiotherapy, Oncofertility, Azoospermia, Fatherhood

## Abstract

**Background:**

In 15–49 years-old men, the main cancers are testicular cancer (TC) and lymphomas (L): freezing of ejaculated sperm is primarily used for male fertility preservation (FP) before cancer treatment.

Our objective was to analyze the French FP rate in 15–49 years-old men diagnosed with TC or L in 2018.

We designed a national descriptive cross-sectional study of sperm banking rate in men with a diagnosis of TC, Hodgkin L (HL) or non-Hodgkin L (NHL).

From the French National Cancer Institute (INCa) 2018 data, we extracted the estimated incidence of TC and L in metropolitan France. From the 2018 activity report of CECOS network (Centers for Study and Banking of Eggs and Sperm), we extracted the number of men with TC or L who banked ejaculated sperm. We estimated the proportion of 15–49 years-old men diagnosed with TC or L who banked sperm.

**Results:**

Among 15–49 years-old men, INCa estimated 38,048 new cancer diagnoses in metropolitan France in 2018: 2,630 TC and 3,913 L (943 HL and 2,970 NHL). The CECOS network provided data from 26/27 metropolitan centers (96% response rate): 1,079 sperm banking for men with TC, 375 for HL and 211 for NHL.

We estimated that the 2018 sperm banking rate in France was 41% for TC, 40% for HL, and 7% for NHL.

**Conclusions:**

To our knowledge, our paper is the first cross-sectional study with multicenter and national data analyzing FP rate in cancer men: it suggests an efficient pathway for men to FP before cancer treatment, compared to previously published studies. Although sperm banking rate in 15–49 years-old men could definitely be improved, further studies should evaluate the information given to patients before gonadotoxic treatments, the factors associated with the absence of sperm banking and whether this lack of referral induces a loss of chance for these men.

## Résumé

**Contexte:**

Chez les hommes de 15 à 49 ans, les principaux cancers sont le cancer du testicule (CT) et les lymhomes (L): la congélation de spermatozoïdes éjaculés est utilisée en première intention pour leur préservation de fertilité (PF) avant traitement du cancer.

Notre objectif était d’analyser le taux de PF chez les hommes de 15 à 49 ans diagnostiqués avec un CT ou un L en 2018 en France.

Nous avons réalisé une étude nationale transversale descriptive du taux de congelation de spermatozoïdes chez les hommes âgés de 15 à 49 ans diagnostiqués avec un CT, un L de Hodgkin (LH) ou un L non-Hodgkinien (LNH).

A partir des données de l’Institut National du Cancer (INCa) de 2018, nous avons extrait l’incidence estimée de CT et de L en France métropolitaine. A partir des données du bilan d’activité 2018 de la Federation Française des CECOS (Centre d’Etude et de Conservation des Oeufs et du Sperme), nous avons extrait le nombre d’hommes avec un CT ou un L qui ont congelé leurs spermatozoïdes. Nous avons enfin estimé la proportion d’hommes de 15 à 49 ans diagnostiqués avec un CT ou un L qui ont congelé leurs spermatozoïdes.

**Résultats:**

Chez les hommes de 15 à 49 ans, l’INCa a estimé en 2018 38 048 nouveaux cas de cancers diagnostiqués en France métropolitaine en 2018: 2 630 CT et 3 913 L (943 LH et 2 970 LNH). Le réseau des CECOS a produit les résultats issus de 26/27 centres métropolitains (taux de réponse de 96%): 1 079 congélations de sperme pour des hommes atteints de CT, 375 pour LH et 211 pour LNH.

Nous avons estimé que le taux de congelation de spermatozoïdes de 2018 en France était de 41% pour le CT, 40% pour le LH et 7% pour le LNH.

**Conclusions:**

A notre connaissance, notre travail est la première étude transversale multicentrique de données nationales analysant le taux de PF chez les hommes atteints de cancer: il suggère un parcours patient efficace pour la PF des hommes avant traitement d’un cancer, par rapport aux études précédemment publiées. Bien que le taux de PF chez les hommes puisse certainemen être amélioré, des études futures devraient évaluer l’information donnée aux patients avant traitement gonadotoxique, les facteurs associés à l’absence de PF et si le défaut d’adressage au CECOS induit un perte de chance pour ces hommes.

**Mots-clés:**

Chimiothérapie, Radiothérapie, Oncofertiité, Azoospermia, Paternité

## Background

For the past 30 years, the overall number of new cancer cases in France has been increasing every year [1]. In men of reproductive age, the main cancers are testicular cancer (TC) and lymphoma (L) [2, 3].

Oncology management has evolved into a multidisciplinary initiative focused on the patient's overall survival and quality of life. Indeed, in France the net survival rate for a man diagnosed with cancer in 2015 was 94% for testicular cancer and 88% for Hodgkin lymphoma (all ages from 20 to 60) [4]. Fertility preservation (FP) is even more important when patients are young, and the conditions have a favourable long-term prognosis, as fertility is an essential part of quality of life in cancer survivors.

The gonadotoxic impact of anticancer drugs (chemotherapy, radiotherapy and pretransplantation stem cell conditioning) is increasingly well documented: alkylating agents (cyclophosphamide, chlorambucil, busulfan, procarbazine, high-dose platinum salts, etc.) are the anticancer agents with the most severe gonadotoxic effects [5], and the impact of chemotherapies on spermatogenesis is dose- and individual dependent. These treatments can lead to quantitative alteration (with a risk of sterility) and/or to qualitative alteration (DNA damage/aneuploidy) of spermatogenesis [6–9]. Furthermore, the spermatogenesis recovery rate is variable and multifactorial, depending on the type of cancer and the type of gonadotoxic treatment [10–12] as well as the patient fertility status.

In this context, sperm banking has been routinely used since the 1970s to preserve male fertility. Freezing of ejaculated sperm is primarily used to preserve fertility in patients before starting oncologic treatment [11]. In France, access to fertility preservation (FP) is guaranteed by the bioethics law: "Any person who is to undergo treatment that may alter his or her fertility has access to information concerning the possibilities of gamete or germinal tissue preservation”. When conservation is performed within the context of a life-threatening pathology, the patient receives specific and targeted information [13]. Patients with banked sperm are contacted annually to extend or not their storage, which is possible until death (no post-mortem use allowed in France). FP is performed in a public or private establishment or laboratory specifically accredited for this activity by the Regional Health Agencies (ARS) and the Biomedicine Agency. FP is fully reimbursed by health insurance. In 1973, a public gamete conservation network was created: the French national sperm banking network CECOS (Centre for the Study and Conservation of Human Eggs and Sperm). CECOS centres include a multidisciplinary team (physiologists, embryologists, psychologists, midwives, geneticists, and technicians) and a specialized cryobiology platform; thirty of the present 31 CECOS centres are linked to a public university hospital. One of the main missions of CECOS is to enable FP for patients before any cancer or non-cancer treatment or circumstance presents a risk for future fertility [14].

Nevertheless, a French survey in 4,349 post-cancer treatment male (< 60 years old) and female (< 40 years old) patients showed in 2017 that although 37% of male cancer patients had a parental project at cancer diagnosis, 2/3 of them were not informed about FP before treatment, and only 16% of them banked sperm before treatment [15]. Moreover, a national study from CECOS network found that the frequency of the recourse to FP for adolescents and young adults (AYA), while increasing between 1973 and 2007, was heterogenous in the French territory [16].

Even if male FP (MFP) is guaranteed by bioethics law [13], the distribution of MFP activity among the various French accredited centres as well as the proportion of men of reproductive age with TC or L who benefit from sperm freezing before gonadotoxic treatment remain unknown. Therefore, the aim of this study was to analyse the French activity of MFP before treatment of testicular cancer or lymphoma.

## Methods

We conducted a national descriptive cross-sectional study of 2018 data concerning sperm banking in pubertal males with a diagnosis of TC, HL and NHL, i.e. the more frequent cancers in 15–49 years-old male adults. This age range is not a CECOS network policy, as no lower nor upper age limit for fertility preservation exists (lower and upper age limits are only for ART use) [16].

### Evaluation of the French incidence of new cancer cases

Every year since 1990, the French National Cancer Institute (INCa) has collected all new cancer cases in the Francim network cancer registries and derived an estimate of cancer incidence and mortality in metropolitan France. The Francim network registries cover between 19 and 22 departments (county-sized administrative divisions), the number of departments depending on the cancer studied [17].

The latest available data are for 2018 (July 2019 report (1)) and are freely available as two PDF files (https://www.santepubliquefrance.fr/maladies-et-traumatismes/cancers/cancer-du-sein/documents/rapport-synthese/estimations-nationales-de-l-incidence-et-de-la-mortalite-par-cancer-en-france-metropolitaine-entre-1990-et-2018-volume-1-tumeurs-solides-etud), Volume 1: solid tumours and Volume 2: haematological malignancies were used for our analysis. The number of cases and the incidence rate are given for each age group: the first group from 0 to 14 years and then every 5 years until 95 years and older.

We selected from the 2018 data; men aged 15 to 49 years (corresponding to men of reproductive age in France) [18] diagnosed with TC, L and NHL. The incidence of these cancers and their distribution according to the age of the patients were derived.

### Evaluation of male fertility preservation (MFP) activity

#### Distribution of MFP activity between the different types of French accredited centres

Every year, the French Biomedicine Agency (Agence de la Biomédecine) collects data on fertility preservation activity from all accredited (private or public) centres in France. The MFP data are divided into three parts: i) sperm conservation activity with a medical indication (cancer or other); ii) sperm conservation before assisted reproductive technique (collection failure, personal convenience); iii) sperm conservation from surgical samples (epididymal, deferential, testicular). The data from the 2018 activity were published in 2020 in the form of a medical and scientific report [19] in open access (https://rams.agence-biomedecine.fr/preservation-de-la-fertilite). We selected the part concerning sperm conservation activity with a medical indication, which provides the number and type of centres involved (private centres, centres of the CECOS network) and the number of new sperm conservations (number of patients) in 2018.

#### Activity of MFP before treatment of testicular cancer (TC) or lymphoma (L) in the CECOS network

Every year, the French CECOS network collects from all its centres the annual activity report of fertility preservation activities. We requested permission from the presidency of the French CECOS network and obtained an agreement to analyse the activity data in an Excel File for the year 2018.

The data from the CECOS network include, by centre, the following: i) The number of MFPs before cancer treatment according to the type of cancer: haematological (HL, NHL, leukaemias, other haematological cancers), urological (TC, bladder, prostate, kidney, other urological cancer), other (digestive, bone, brain, lung, melanoma, other). (ii) The type of cryopreserved samples (ejaculated sperm, surgically retrieved sperm, and testicular tissue). (iii) The number of MFPs for noncancerous conditions, with their indications.

We extracted data concerning ejaculated sperm freezing before treatment of TC, HL and NHL in metropolitan centres of the CECOS network.

### Data analysis

We first estimated the distribution of MFP activity by sperm freezing in metropolitan France in 2018 in private and CECOS centres by analysing Biomedicine Agency data.

Then, we estimated the proportion of patients of reproductive age with TC or L who cryopreserved sperm by comparing the estimated national incidence in 2018 of these new cancers (INCa data) with the number of MFPs by sperm freezing performed in the CECOS centres for these indications during the same period.

Because our study was an analysis of the CECOS network’s annual operating report and public data from the National Institute of Cancer and Biomedicine Agency, it did not require any Institutional Review Board authorization.

## Results

### Cancer incidence in men of reproductive age in France in 2018

Among men aged 15–49 years, INCa estimated 38,048 new cancer diagnoses in metropolitan France in 2018: 2,630 new cases of TC and 3,913 new cases of lymphomas (943 HL and 2,970 NHL). These 6,543 new cases of TC, HL and NHL represent 55% of the 11,994 new cases of the main cancers of this age group (TC, HL, NHL, skin melanoma, lung, colon-rectum, kidney and central nervous system cancers).

### Male fertility preservation activity in France in 2018

- Data from the Biomedicine Agency report were provided by 49/55 centres specifically accredited for gamete and/or germinal tissue preservation: 21/26 fertility centres and 28/29 CECOS centres. The MFP activity for medical indication (cancer or other) was 5,636 new sperm conservations, of which 5,062 (90%) were performed within the CECOS network.
- Data from the CECOS network were provided by 26/27 centres in metropolitan France existing in 2018 (the 2 overseas centres were excluded, and 2 additional CECOS centres were created since 2018). Sperm were banked for 1,079 patients with TC, 375 with HL and 211 with NHL. These 1,665 TC, HL and NHL patients represent 65% of the 2,537 patients with sperm banking(s) for the main cancers of this age group (TC, HL, NHL, skin melanoma, lung, colon-rectum, kidney and central nervous system cancers).

### Proportion of men of reproductive age diagnosed with cancer in 2018 who banked sperm

The proportions of men of reproductive age with TC or L in metropolitan France who banked ejaculated sperm in 2018 in the CECOS network centres are presented in Table 1.

**Table 1:** Proportion of 15–49 years-old men with the 3 most common types of cancers of this age range, who banked ejaculated sperm in 2018 in France

	**Estimated number of new cases**	**Number of FP**	**Rate of FP**
**Testicular cancer**	2,630	1,079	41%
**Lymphoma**	3,913	586	15%
Hodgkin lymphoma	943	375	40%
Non-Hodgkin lymphoma	2,970	211	7%
**Total**	6,543	1,665	25%

*FP* Fertility preservation by ejaculated sperm banking(s) for one man

Estimated number of new cases: extracted from 2018 data of National French Institute of Cancer

Number of FP: extracted from 2018 national data of the French Federation of CECOS (representing 90% of sperm banking activity for medical indication in France)

## Discussion

To our knowledge, we present the first cross-sectional study analysing national data about the rate of male fertility preservation (MFP) in men of reproductive age newly diagnosed with testicular cancer (TC), Hodgkin lymphoma (HL) or non-Hodgkin lymphoma (NHL). Our results are based on the annual incidence of new cancer cases estimated by the French National Cancer Institute (INCa) and on the annual activity report of the CECOS network, which performs 90% of French sperm banking activity for medical indication. We showed that 40% of the 3,573 15–49 years-old men diagnosed with TC or HL in 2018 received sperm banking, following guidelines of the National Institute of Cancer [20].

Studies analysing male fertility preservation (MFP) rate before TC or HL treatment are rare. Furthermore, in a systematic review, Valipour and al., recently showed a major heterogeneity in the designs of studies previously published on this question [21]. Indeed, a very wide range of MFP rates before TC or L treatment are described, which is mainly related to a variable definition of MFP rate: studies analysing the rate of sperm banking in cancer patients who were informed of MFP, generally report high MFP rates (73% in 166 TC patients [22], 69% in 35 TC patients [23]). Most of studies analysing the rate of sperm banking in all cancer patients (informed or not about MFP, like in our study) are retrospective studies performed in cancer survivors. Concerning TC patients, only 5 studies with such a design are available: Brydoy et al., described a 23% MFP rate in 326 TC survivors [24], Sonennburg et al., 31% in 200 TC survivors [25], Uçar et al., 43% in 110 TC survivors from one centre [20] and Selter et al., 12% in 2,610 patients (aged 18 to 40 years) who underwent TC surgery, recorded in MarketScan database [26]. Ping et al., analysed a sub-population of TC survivors (men remained childless, *n* = 96) and described a 18% fertility preservation rate [27]. Concerning HL patients, only 3 studies analysing the MFP rate in a population of cancer patients informed or not about MFP are available in the literature: Fitoussi et al., described a 30% MFP rate in 316 HL survivors [28], Van der Kaaij et al., 40% in 902 HL survivors (with a 50% participation rate of the target population [29]) and Boltezar et al., 79% in 47 HL survivors diagnosed before 40 years-old in one centre [30]. Concerning NHL, no data from studies with such a design are available to our knowledge.

Based on these reports, we observe that although the 40% sperm banking rate in 15–49 years-old men diagnosed with TC or HL in 2018 in France could definitely be improved, it is one of the highest in multicentre and large population-based studies, which suggests to focus on the organisation of MFP pathway in France.

### Organisation of pathway for male fertility preservation before cancer treatment

The report of the Biomedicine Agency on FP activity showed that 90% of MFP procedures with medical indication (cancer or other) are performed in the CECOS network centres. This result confirms the data assessed by a previous national study [31] and shows that the patient’s pathway for MFP before cancer treatment in France is mainly oriented towards CECOS network centres.

The missions of the CECOS network are to coordinate practices between the different CECOS centres in order to harmonize the quality of patient care, to promote research, to improve the quality of practices, to support the centres in their decision-making, to promote exchanges between centres and to inform the public of the CECOS missions (https://www.cecos.org/) [16]. One of the strengths of the CECOS network is the establishment of multidisciplinary platforms, including oncologists, reproductive biologists, nurses, psychologists and sexologists: covering the whole French territory, these platforms play a key role in the coordination of care. They not only improve the transmission of information between oncologists and CECOS centres to facilitate the organisation of fertility preservation but also improve the follow-up of patients after cancer [32, 33], which is crucial to improve the quality of life of cancer survivors; thus, erectile dysfunction linked to hypogonadism may appear 3 to 5 years after the end of treatment and require appropriate management [34, 35].

### Interest in oncofertility platforms

The INCa (National French Institute of Cancer) published an update of recommendations for fertility preservation in the field of cancer in 2021. The observation of these recommendations by oncologists should increase the referral of cancer patients to fertility preservation platforms [36].

The French cancer plan has led to the development of several regional cancer networks covering all of France. These cancer networks contribute to good coordination between services, to the regional organisation of cancer care and to the harmonization of practices [37] between the various health professionals and patients around cancer. A study on the benefits of the regional network in southeastern France found a significant improvement in the information given to patients before the start of gonadotoxic treatment and an improvement in the proportion of patients referred for postcancer sperm monitoring. Many physicians used this platform as a source of information with a good level of satisfaction [38].

Other countries are setting up comparable organisations, such as the Fertiprotekt network [39], created in 2006, and the Oncofertility Consortium (OC), created in 2007, which provides a scientific, intellectual and financial resource to help better care for young cancer patients. This organisation currently has 19 countries (including the United States, Canada, Brazil, Australia, Germany, Italy, England, India, China, Russia, Japan…) and has formed the National Physicians Cooperative, which represents more than 60 centres in the United States offering oncofertility services to men and women, as well as 19 centres focused on paediatric patients [40]. The OC makes materials available (oncofertility.northwestern.edu) and promotes cross-country interaction, comparison of practices, and field experiences. With the establishment of a multidisciplinary platform, the rate of male FP in cancer increased almost sixfold, from 3.3% FP between 2011 and 2016 to 19.3% FP between 2016 and 2018, all indications combined [41].

### Factors associated with referral rate for sperm banking in cancer men

We observed a higher MFP rate in TC and HL (41% and 40% of patients, respectively) than in NHL (7% of patients). Testicular cancer requiring chemotherapy usually receives a bleomycin/etoposide/cisplatin regimen, and non-Hodgkin lymphoma usually receives a cyclophosphamide/doxorubicin/vincristine/prednisone regimen, which both induce an intermediate risk of sterility of 25–75%. Hodgkin lymphoma usually receives Adriamycin / bleomycin / vinblastine / dacarbazine regimen, which is associated with a much lower risk of infertility (< 25%); nevertheless, if a lymphoma recurrence occurs, an autologous stem cell transplant is then required, with a very high risk of sterility (> 75%)(carmustine / etoposide /cytosine-arabinosi / melphalan conditioning regime after a rituximab / cisplatine / cytosine-arabinosine / dexamethasone or rituximab / ifosfamide / carboplatine / etoposide chemotherapy) [12, 42]. In TC, after orchidectomy, the risk of sterility increases in men with only one testis (if this only testis is damaged). Overall, the various gonadotoxicities of the usual treatments of TC, HL and NHL do not seem to be associated with the rate of MFP assessed in our study.

The maximum incidence of TC is between 30–34 years old, of HL is between 25–29 years old and of NHL is approximately 50 years old. A higher mean age in NHL patients versus TC and HL patients could be associated with a lower referral rate. Indeed, the chance for a cancer patient to be referred to an FP platform is strongly linked to fertility knowledge and the usual practice of medical staff: patients with advanced age or already parented are significantly less likely to be referred than others, and patients aged 20–34 years are the most referred [32, 43–45]. Clinicians may also be uncomfortable discussing MFPs with adolescents, although highly successful MFPs have been described in 11- to 14- and 15- to 18-year-old adolescents [16, 46]. On the other hand, patient acceptance of MFP may also be influenced by his age: Scott et al. recently described a significantly decreased uptake of sperm banking in 35–45 years old patients with TC versus patients younger than 35 or older than 45 years old [47].

Lack of time could be associated to non-referral of a sizeable proportion of NHL patients: a British study suggested that 41% of NHL patients aged < 50 years-old were diagnosed via emergency admissions [48] condition which often requires an urgent treatment. An Italian survey in 152 hematologic centres assessed important issues regarding access delay for fertility consultation and sperm banking (up to 40 days) [49]. For patients with TC, lack of time should not influence MFP, as sperm banking can be performed effectively after orchidectomy. Nevertheless, especially in seminomas, sperm banking should be organised before orchidectomy [50].

Another factor could be a lack of information in medical teams and oncologists about FP techniques and indications, as suggested in a French study [51]. Patients are more likely to bank sperm when they have received information (risks and options) about MFPs [52]. Studies show that the more patients are informed, the higher the preservation rate: 73% of informed patients banked sperm for TC [22], 79% of informed patients banked sperm for HL [30]. In the United Kingdom and the United States, an estimated 40–50% of patients received information [23, 53, 54].

The difference in MFP rates across countries may be due to the way this procedure is funded. In the United States, financial barriers are reported, with decision-making based on the social level of the patient [55]. The average cost of semen analysis and storage for three years is $1,000 to $1,500, which varies by centre (Family building options for men: http://livestrong.org/Fertility). Insurance varies, but most do not cover the cost. Conversely, in France, MFP is fully reimbursed by the health insurance system (freezing procedure as well as conservation until 59 years old).

Finally, psychosocial factors related to personal convictions (lack of interest) or to the marital situation (couple with children, single or gay patient) have also been mentioned [25, 55, 56].

Our study did not take into account patients who were informed of the possibility of FP but who did not wish to bank sperm, who were unable to bank sperm (pretreatment azoospermia, sperm collection failure, no-show…), or who underwent oncological testicular sperm extraction (onco-TESE). The estimated rate of non sperm banking in cancer patients referred to CECOS network centres is 10% [57].

### Strengths and limitations

A strength of our study is the very high participation rate of CECOS centres covering the French territory and the use of official national data on cancer incidence and FP activity. To our knowledge, our study is the first with national data and is also the only one with a cross-sectional design; this avoids the selection bias of retrospective studies, which are based only on volunteer cancer survivors.

One limitation of our study is that the Biomedicine Agency data (number of sperm banking for medical indication) did not distinguish between MFP performed for cancer and for another medical indication (the latter estimated to 20% of FP for medical indication, unpublished data of FP activity in CECOS network), which could lead to an overestimation of MFP activity before cancer treatment. The Biomedicine Agency annual data did not either provide the indications of surgical sperm extractions (before ART or before cancer treatment). Consequently, we could not include cancer patients whose fertility was preserved by banking of surgically retrieved sperm (onco-testicular sperm extraction, onco-TESE), which could induce and underestimation of MFP activity before cancer treatment.

Another limitation is that the report of the CECOS network did not provide the number of patients referred to the CECOS for whom there was no banking of ejaculated sperm (collection failure, azoospermia, onco-TESE). The data from the CECOS network did not either specify the patients’ age; consequently, we could not exclude sperm banking performed in patients aged less than 15 years or more than 49 years. Nevertheless, the incidence of these two types of cancer is reduced in these age brackets; however, this could also generate an overestimation of the MFP rate in cancer men aged 15 to 49 years, and we could not analyse the relations between cancer patient age and the MFP rate.

Due to the suboptimal precision of national French data on TC and L incidence and on sperm banking activity for cancer, our assessment could overestimate the rate of men with TC or L who banked sperm before cancer treatment. The failure to provide information on MFP to patients receiving cancer treatment could lead to impossibility of biological parenthood in patients with lasting posttreatment azoospermia; if the man was not given information before treatment, the oncologist could be held responsible.

It would have been very interesting to compare referral rates between the various French regions, and also to include overseas regions in our study; unfortunately, the data from the National Cancer Institute (INCa) were not comprehensive, several centres of the CECOS network were in a region with no cancer data available and INCa data did not include overseas regions. However, INCa data are the only data available about the national incidence of new cancer cases in France.

## Conclusions

In this cross-sectional study, we showed that 40% of men aged 15–49 years with testicular cancer or Hodgkin lymphoma in France received sperm banking before cancer treatment. To our knowledge, our paper is the first with multicentre national data of a large population and this fertility preservation rate is one of the highest. It suggests an efficient pathway for men to fertility preservation before cancer treatment compared to previously published studies. Nevertheless, this fertility preservation rate is not optimal and could definitely be improved, which would require better prevention and andrology assessment, better information in patients, and better organisation and training in health structures and practitioners. Further studies should evaluate the information given to patients before gonadotoxic treatments in large prospective/transversal studies, identify the factors associated with the absence of referral for sperm banking and evaluate whether the lack of sperm banking induces a loss of chance for these men.

## Data Availability

The datasets used and/or analysed during the current study are available from the corresponding author on reasonable request.

## Abbreviations

CECOS: Centre for the Study and Conservation of Eggs and Sperm (Centre d’Etude et de Conservation des Oeufs et du Sperme)
FP: Fertility preservation
HL: Hodgkin lymphoma
INCa: French National Cancer Institute (Institut National du Cancer)
L: Lymphoma
MFP: Male fertility preservation
NHL: Non-Hodgkin lymphoma
OC: Oncofertility Consortium
TC: Testicular cancer
TESE: Testicular sperm extraction
