# Correction: Efficient pathway for men fertility preservation in testicular cancer or lymphoma: a cross-sectional study of national 2018 data

**DOI:** 10.1186/s12610-024-00221-6

**Published:** 2024-02-05

**Authors:** Ségolène Prades, Sarah-Lyne Jos, Jacqueline Saïas-Magnan, Louis Bujan, Florence Eustache, Oxana Blagosklonov, Eric Lechevallier, Florence Brugnon, Vanessa Loup-Cabaniols, Dorian Bosquet, Marie Prades, Bérengère Ducrocq, Céline Chalas, Sandrine Giscard-d’Estaing, Anne Mayeur, Isabelle Koscinsky, Françoise Schmitt, Aline Papaxanthos-Roche, Marius Teletin, Emmanuelle Thibault, Damien Beauvillard, Sophie Mirallie, Béatrice Delepine, Annie Benhaim, Pascale May-Panloup, Ségolène Veau, Cynthia Frapsauce, Patricia Fauque, Régis Costello, Nathalie Rives, Catherine Metzler-Guillemain, Jeanne Perrin, CECOS network

**Affiliations:** 1 CECOS/Laboratory of Reproductive Biology, La Conception University Hospital, 13385 Marseille, France; 2 DEFE (Développement Embryonnaire, Fertilité, Environnement) INSERM, Universités Montpellier Et Toulouse. 3, CECOS Hôpital Paule de Viguier, CHU de Toulouse, 1202 Toulouse, France; 3 CECOS, Site Jean Verdier, Hôpitaux Universitaires Paris Seine-Saint-Denis, Bondy, France; 4 Genomics, Epigenetics and Physiopathology of Reproduction, Institut Cochin, Inserm U1016, Paris, France; 5 Service de Biologie Et Medecine de La Reproduction, Cryobiologie-CECOS, CHRU Jean Minjoz, Besançon, France; 6 Service d’Urologie Et Transplantation Rénale, Aix-Marseille Université, Assistance Publique Hôpitaux de Marseille, Marseille, France; 7 1240 IMOST, INSERM, Clermont Ferrand, France; 8 Service AMP CECOS, CHU Clermont Fer- Rand, Clermont Ferrand, France; 9 CECOS Languedoc Roussillon, MONTPELLIER Hôpital Arnaud de Villeneuve, Montpellier, France; 10 Service de Médecine Et Biologie de la Reproduction – CECOS-CHU Amiens Picardie - Site Sud, Amiens, France; 11 Service de Biologie de La Reproduction-CECOS, Hôpital Tenon (AP-HP), Sorbonne-Université, 75020 Paris, France; 12 Institut de Biologie de La Reproduction - CECOS Hôpital Calmette, CHU de Lille, Lille, France; 13 Labora- Toire de Biologie de La Reproduction-CECOS, Assistance Publique-Hôpitaux de Paris. Centre Université Paris-Cité, GHU Cochin, Paris, France; 14 Biologie de La Reproduction, U1208, Hospices Civil de Lyon, HFME, Inserm, Bron, France; 15 Faculté de Médecine Lyon Sud, Université Claude Bernard, Oullins, France; 16 Reproductive Biology Department, CECOS, Paris-Saclay University, Antoine-Béclère Hospital, APHP, Clamart, France; 17 NGERE (Nutrition Géné- Tique Et Exposition Aux Risques Environnementaux) INSERM 1256, Université de Lorraine, 10 Avenue de La Forêt de Haye, 54505 Vandoeuvre Les Nancy, France; 18 Laboratoire de Biologie de La Reproduction, Hôpital Saint Joseph 26 Boulevard de Louvain, 13008 Marseille, France; 19 CECOS ALSACE Mulhouse Groupe Hospitalier, de La Région de Mulhouse Et Sud Alsace, Mulhouse, France; 20 Biology of Reproduction-CECOS Laboratory, University Hospital of Bordeaux, Bordeaux, France; 21 Institut de Génétique Et de Biologie Molécu- Laire Et Cellulaire (IGBMC), Université́ de Strasbourg, France-LBDR-CECOS, Hôpitaux Universitaires de Strasbourg (HUS), Strasbourg, France; 22 Labora- toire de Biologie de la Reproduction – CECOS Hôpital L’Archet 2 - CHU de Nice, Nice, France; 23 CHU Brest, AMP CECOS, Brest, France; 24 Service de Médecine Et Biologie de La Reproduction, CHU, Nantes, France; 25 Service de Biologie de La Reproduction Reims - Pôle de Biologie Médicale Et Pathologie, Reims, France; 26 Service de Biologie de La Reproduction Coordinatrice Clinico-Biologique du Centre d’AMP du CHU de Caen Pôle de Biologie-CHU, Caen, France; 27 Biologie de La Reproduction, Centre Hospitalier Universitaire & Univ Angers, INSERM, CNRS, MITOVASC, Equipe MitoLab, SFR ICAT, 49000 Angers, France; 28 Service de Biologie de La Reproduction-CECOS, CHU Rennes - Hôpital Sud, Rennes, France; 29 Service de Médecine Et de Biologie de La Reproduction-CECOS, CHRU Bretonneau, Tours, France; 30 Burgundy University, INSERM 1231, Dijon, France; 31 Dijon University Hospital, Biology of Reproduction-CECOS Labora- Tory, Dijon, France; 32 Service d’Hématologie Et Thérapie Cellulaire, Hôpital La Conception, Assistance Publique Des Hôpitaux de Marseille, 147 Boulevard Baille, 13005 Marseille, France; 33 NorDiC UMR 1239, Team “Adrenal and Gonadal Pathophysiology”, Biology of Reproduction-CECOS Laboratory, Univ Rouen Normandie, Inserm, Rouen University Hospital, Rouen, France; 34 Aix Marseille Univ, INSERM, MMG, UMR_S1251, Marseille, France; 35 Aix Marseille Univ, Avi- Gnon Université, CNRS, IRD, IMBE, Marseille, France

**Correction: Basic Clin Androl. 33, 35 (2023)**

**https://doi.org/10.1186/s12610-023-00209-8**

Following publication of the original article [1], the authors reported an error in affiliation 7. The correct affiliation is: 1240 IMOST, INSERM, Clermont Ferrand, France.

The original article [1] has been corrected.
